# Residue-Specific Annotation of Disorder-to-Order Transition and Cathepsin Inhibition of a Propeptide-Like Crammer from *D. melanogaster*

**DOI:** 10.1371/journal.pone.0054187

**Published:** 2013-01-21

**Authors:** Tien-Sheng Tseng, Chao-Sheng Cheng, Shang-Te Danny Hsu, Min-Fang Shih, Pei-Lin He, Ping-Chiang Lyu

**Affiliations:** 1 Institute of Bioinformatics and Structural Biology, National Tsing Hua University, Hsinchu, Taiwan; 2 Institute of Biological Chemistry Academic Sinica, Taipei, Taiwan; 3 Graduate Institute of Molecular Systems Biomedicine, China Medical University, Taichung, Taiwan; Instituto de Tecnologica Química e Biológica, UNL, Portugal

## Abstract

*Drosophila melanogaster* crammer is a novel cathepsin inhibitor involved in long-term memory formation. A molten globule-to-ordered structure transition is required for cathepsin inhibition. This study reports the use of alanine scanning to probe the critical residues in the two hydrophobic cores and the salt bridges of crammer in the context of disorder-to-order transition and cathepsin inhibition. Alanine substitution of the aromatic residues W9, Y12, F16, Y20, Y32, and W53 within the hydrophobic cores, and charged residues E8, R28, R29, and E67 in the salt bridges considerably decrease the ability of crammer to inhibit *Drosophila* cathepsin B (CTSB). Far-UV circular dichroism (CD), intrinsic fluorescence, and nuclear magnetic resonance (NMR) spectroscopies show that removing most of the aromatic and charged side-chains substantially reduces thermostability, alters pH-dependent helix formation, and disrupts the molten globule-to-ordered structure transition. Molecular modeling indicates that W53 in the hydrophobic Core 2 is essential for the interaction between crammer and the prosegment binding loop (PBL) of CTSB; the salt bridge between R28 and E67 is critical for the appropriate alignment of the α-helix 4 toward the CTSB active cleft. The results of this study show detailed residue-specific dissection of folding transition and functional contributions of the hydrophobic cores and salt bridges in crammer, which have hitherto not been characterized for cathepsin inhibition by propeptide-like cysteine protease inhibitors. Because of the involvements of cathepsin inhibitors in neurodegenerative diseases, these structural insights can serve as a template for further development of therapeutic inhibitors against human cathepsins.

## Introduction

Cysteine proteases, which are responsible for the processes of intra- and extra-cellular protein degradation and turn-over [1], are widely distributed in bacteria, plants, invertebrates, and vertebrates [2]. The papain family of cysteine proteases is one of the largest groups of proteases. This family includes lysosomal cathepsins B, C, H, L, and S, and the more recently described cathepsins F, K, O, V, W, and X [3]. Cathepsins are associated with various clinical conditions such as bone disorder pycnodysostosis [4], bone resorption [5], arthritis [6], and tumor metastases [7]. Recent research has shown that cathepsin activity also regulates the long-term memory formation of *Drosophila melanogaster* [8]. The relationship between cathepsins and a number of neurodegenerative diseases, such as Parkinson’s and Alzheimer’s diseases, is also emerging as an important subject [9]–[11]. Given the involvement of cathepsins in many physiological processes, they have become attractive targets for drug development and other therapeutic applications [3], [12]–[21].

Research over the past two decades has reported several propeptide-like cysteine protease inhibitors [22]–[27]. Other research has determined their sequences and structural homology with respect to the proregions of their targeted proteases [28]. Propeptide-like cysteine protease inhibitors have the ability to inhibit cathepsins, and many researchers have investigated their functional and structural properties [22], [24]–[29]. One of these inhibitors, crammer from *D. melanogaster*, also known as *Drosophila* cytotoxic T lymphocyte antigen-2 (CTLA-2)-like protein, (D/CTLA-2), is of particular interest [8]. Crammer is a small protein of 79 amino acids that shares approximately 37% of its sequence with other cathepsin proregions and propeptide-like cysteine protease inhibitors [28]. This crammer is involved in the long-term memory formation of *D. melanogaster* [8], [30]. Crammer functions as a strong, competitive inhibitor against CTSL-like cathepsins, and requires a molten globule-to-ordered structure transition to inhibit cathepsins [28]. In addition, we have determined the solution structure of a crammer variant, C72S (a Cys to Ser point mutation at position 72), the first of a propeptide-like cathepsin inhibitor that has been reported in the literature. Under acidic conditions (pH 4.0), C72S behaves like wild type crammer (designated as WT hereafter) which forms a molten globule. At pH 6.0, C72S adopts a structure that resembles the structure of cathepsin-bound WT crammer, encompassing two hydrophobic cores and four salt bridges that may contribute to crammer’s folding stability. The structure of crammer highly resembles that of human cathepsin propeptides, suggesting its potential inhibitory activity toward human cathepsins [28].

Multiple sequence alignment of crammer with its homologs indicates that several aromatic residues in the hydrophobic cores and charged residues of the salt bridges in crammer are highly conserved among cathepsin propeptides and cytotoxic T lymphocyte antigen-2α and β (CTLA-2α and 2β). Using site-directed mutagenesis, Wiederanders et al. showed that W28, W31, and W52 of human cathepsin S propeptide are essential for the inhibition of mature cathepsin S, and that the aromatic stacking of these residues stabilizes the core structure of the propeptide domain [31]. Yamamo et al. also showed that W12, W15, and W35 of CTLA-2α play important roles in rat cathepsin L inhibition by maintaining the correct structure of CTLA-2α [32]. Although these studies have shed some light on the importance of tryptophan residues in cathepsin inhibition, little is known about the roles of these conserved residues within the hydrophobic cores and salt bridges of the propeptides and the propeptide-like cysteine protease inhibitors in the context of protein folding and cathepsin inhibition.

Based on structural information obtained previously [28], this study systematically examines the contributions of individual residues in crammer. This study uses alanine scanning to investigate the structure-function relationship of the hydrophobic cores and salt bridges in the disorder-to-order transition and CTSB inhibition. Far-UV CD, intrinsic fluorescence, and multidimensional heteronuclear NMR spectroscopies are used to examine the effects of alanine substitution on the structures and folding stabilities of crammer variants. Comparative enzymatic activities provide valuable information on the individual amino acids and their abilities to inhibit cathepsins and folding transition. These structural and functional insights present valuable data for the future development of therapeutic protease inhibitors.

## Materials and Methods

### Materials

L-trans-epoxysuccinyl-leucylamido (4-guanidino) butane (E-64) and Z-Phe-Arg 7-amido-4-methylcoumarin hydrochloride (ZFR-AMC) were obtained from Sigma-Aldrich (St. Louis, MO). β-Mercaptoethanol and chloramphenicol were supplied by Merck Research Laboratories (Rahway, NJ). Ampicillin, isopropyl β-D-1-thiogalactopyranoside (IPTG), and dithiothreitol (DTT) were purchased from MDBio Inc. (Taipei, Taiwan). Ethylenediaminetetraacetic acid (EDTA) was purchased from USB Corporation (Cleveland, OH). Glacial acetic acid was supplied by Echo Chemical Company, Ltd. (Miaoli, Taiwan). All chemicals used were of analytical grade quality.

### Production of Mutant and WT Crammer

WT was cloned into a pAED4 vector [28] as the template for constructing the alanine mutants: D6A, E8A, W9A, Y12A, F16A, Y20A, E24A, R28A, R29A, Y32A, K36A, F46A, W53A, E67A, and C72S. The C72S construct was subsequently used to generate the double mutants: C72S/D6A, C72S/E8A, C72S/W9A, C72S/Y12A, C72S/F16A, C72S/Y20A, C72S/E24A, C72S/R28A, C72S/R29A, C72S/Y32A, C72S/K36A, C72S/F46A, C72S/W53A, and C72S/E67A. All the mutants were generated using the QuickChange PCR-mediated site-directed mutagenesis kit (Stratagene, Amsterdam, The Netherlands). The sequences of the recombinant genes were verified by DNA sequencing (Mission Biotechnology Inc., Taipei, Taiwan). WT and its mutant constructs were transformed into an *E. coli* Rosetta (DE3) strain (Merck, Darmstadt, Germany). The cells were incubated at 37°C in lysogeny broth (LB) [33] containing ampicillin (100 mg/ml) and chloramphenicol (30 mg/ml). When the optical density (OD_600_) of the cell suspension reached 0.7, IPTG was added to the cell culture at a final concentration of 1 mM to induce recombinant protein overexpression. ^15^N-labeled recombinant proteins were obtained from *E. coli* cultures in M9 minimal medium [33] containing 1 g/l of ^15^NH_4_Cl (Cambridge Isotope Laboratories, Andover, MA) [34]. After 3 h of induction, *E. coli* cells were harvested by centrifugation at 6,000× *g* for 20 min, and the resulting cell pellets were lysed with glacial acetic acid. The lysate was subsequently subjected to centrifugation at 30,700× *g* for 20 min, and the supernatant was collected and dialyzed against Milli-Q water at 4°C overnight. A second centrifugation at 30,700× *g* for 20 min was then used to remove any precipitants. All recombinant proteins were purified using a C_18_ semi-preparative column (Nacalai Inc., San Diego, CA) coupled to a 1100 Series reverse-phase high performance liquid chromatography (RP-HPLC) system (Agilent Technologies, Santa Clara, CA). A linear water/acetonitrile gradient (from 29% to 55% acetonitrile over 40****min) was used for protein separation at a flow rate of 1 ml/min. The purified protein fractions were characterized by an Autoflex III MALDI-TOF Mass Spectrometer (Bruker Daltonics Inc., Billerica, MA). Protein concentrations were determined using the Bio-Rad Protein Assay (Bio-Rad, Hercules, CA) with bovine serum albumin as the standard.

### Expression and Purification of *Drosophila* Cathepsin B

The expression and purification of *Drosophila* cathepsin B (CTSB) were performed as described previously [28]. The CTSB construct was transformed into *E. coli* BL21-Gold (DE3) cells (Stratagene, Amsterdam, The Netherlands) and then cultured in LB containing ampicillin (50 mg/ml) at 37°C. When the OD_600_ of the culture reached 0.7, the protein was induced by IPTG (at a final concentration of 1 mM) for 3.5 h. Cells were harvested by centrifugation at 4,000× *g* for 20 min and lysed by sonication. The lysates were further centrifuged at 16,000× *g* for 30 min at 4°C. The overexpressed CTSB existed mainly in the inclusion body and therefore *in vitro* refolding was required to obtain purified CTSB. The supernatant and cell lysate were discarded, and the pellets were dissolved in 40 ml of 6 M guanidine hydrochloride buffer (50 mM Tris/HCl (pH 8.0) 150 mM NaCl, 5 mM EDTA, and 10 mM DTT, 6 M guanidine-HCl). For *in vitro* refolding, the denatured protein solution was diluted into 1 liter of 50 mM Tris/HCl (pH 8.5), 150 mM NaCl, 5 mM EDTA, 10 mM reduced glutathione, 1 mM oxidized glutathione, and 0.5 M arginine and incubated overnight at 4°C with stirring to facilitate refolding. The protein solution was subsequently concentrated and dialyzed against 25 mM NaH_2_PO_4_, pH 7.0, 0.5 M NaCl at 4°C, to yield approximately 50 ml of protein solution**.**

Procathepsin B must be autoprocessed (i.e., the propeptide must be removed prior to activation). The autoprocessing procedure has been described elsewhere previously [35]. Briefly, procathepsin B was activated by adjusting the pH value to 4.5 with glacial acetic acid, followed by the addition of 5 mM EDTA and 5 mM DTT and incubation at 37°C for 1 h. The reactant was then purified using a HiPrep Sephacryl S-100 high resolution gel filtration column with an AKTA prime FPLC system (GE Healthcare, Piscataway, NJ). The running buffer was 100 mM sodium acetate buffer (pH 5.0) containing 1 mM EDTA and 2 mM DTT. The enzyme activity of each elution was confirmed using the cathepsin B-specific substrate, ZFR-AMC [36], [37].

### Inhibition Assay of Cathepsin B

The concentration of active CTSB was determined using the cysteine protease inhibitor, E-64 [38], as described previously [8], [28], [29]. A 70 mg sample of each recombinant protein was dissolved in 10 mM citric acid-sodium phosphate buffer (pH 4.0), to yield a stock protein solution of 0.5 mM. Samples with various protein concentrations were obtained by diluting the stock solution with the same buffer. These samples were subsequently incubated with cathepsin CTSB (75 nM) in 100 mM sodium acetate (pH 5.0) containing 1 mM EDTA and 2 mM DTT. After five minutes, 1.5 µl of 10 mM ZFR-AMC fluorogenic substrate was added to the 798.5 µl CTSB solutions [36], [37]. Residual enzyme activity was measured at 20°C by monitoring the amount of released AMC, by its fluorescence emission at 440 nm (λ_ex_ = 380 nm) [36], [37].

### Far-UV CD and Fluorescence Spectroscopy Experiments

Far-UV CD and fluorescence spectroscopies were performed using previously described procedures [28]. The contents of the protein secondary structure were determined using an Aviv CD spectrometer (Model 202, Aviv Biomedical Inc., Lakewood, NJ). Mutant proteins were dissolved in 10 mM citric acid-sodium phosphate buffer (at pH 4.0 and 6.0) to yield protein concentrations of 30 µM. Far-UV CD spectra (260–190 nm) were acquired at 20°C using a 1-mm path length quartz cuvette. All spectra were averaged over three scans and the data was converted to mean residue ellipticity [θ] [39]. The helical content of individual samples was estimated using the CDNN software [40] and the agadir program (http://agadir.crg.es) and CD spectroscopy was used to determine the protein stability of the mutants at pH 4.0 and 6.0. Thermal denaturation experiments were conducted by monitoring changes in the CD signal at 208 nm between 4°C to 96°C in 2°C increments. The thermodynamic properties of crammer variants were calculated by fitting the unfolding curves with the following equation [41]:
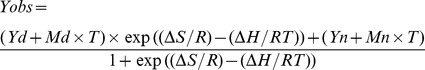
where *Yobs* is the observed CD signal at a given temperature, *T* in Kelvin (K). *Yn* and *Mn* represent the intercept and the slope of the pre-transition straight line, respectively. *Yd* and *Md* represent the intercept and slope of the post-transition straight line, respectively. After curve fitting, enthalpy (ΔH) and entropy (ΔS) of mutants were determined (Table S1). The unfolding free energy change (ΔG_u_) was also deduced on the basis of the Gibbs free energy equation. Moreover, the intrinsic fluorescence of crammer variants was monitored at pH 4.0 and 6.0 using a fluorescence spectrophotometer (model F-7000, Hitachi, Tokyo, Japan). The protein concentrations were 30 µM. The excitation wavelength used was 280 nm and fluorescence emission was monitored between 290 nm and 400 nm.

### Heteronuclear ^1^H-^15^N HSQC NMR Experiments

Uniformly ^15^N-labeled double mutants were buffered in 10 mM citric acid-sodium phosphate buffer with 10% D_2_O (v/v) for heteronuclear NMR studies at pH 4.0 and 6.0. ^1^H-^15^N heteronuclear single quantum correlation (HSQC) spectra were recorded at 25°C using a 600 MHz NMR spectrometer (Bruker Biospin, Kalshruhe, Germany) equipped with a ^1^H/^13^C/^15^N triple resonance room temperature probe. All NMR data were processed using TopSpin (Bruker) and NMRPipe [42], and subsequently analyzed using Sparky software (T.D. Goddard and D.G. Kneller, University of California, San Francisco, www.cgl.ucsf.edu/home/sparky).

## Results

### Inhibition of Drosophila CTSB by the Various Crammer Mutants

**Figure 1:**
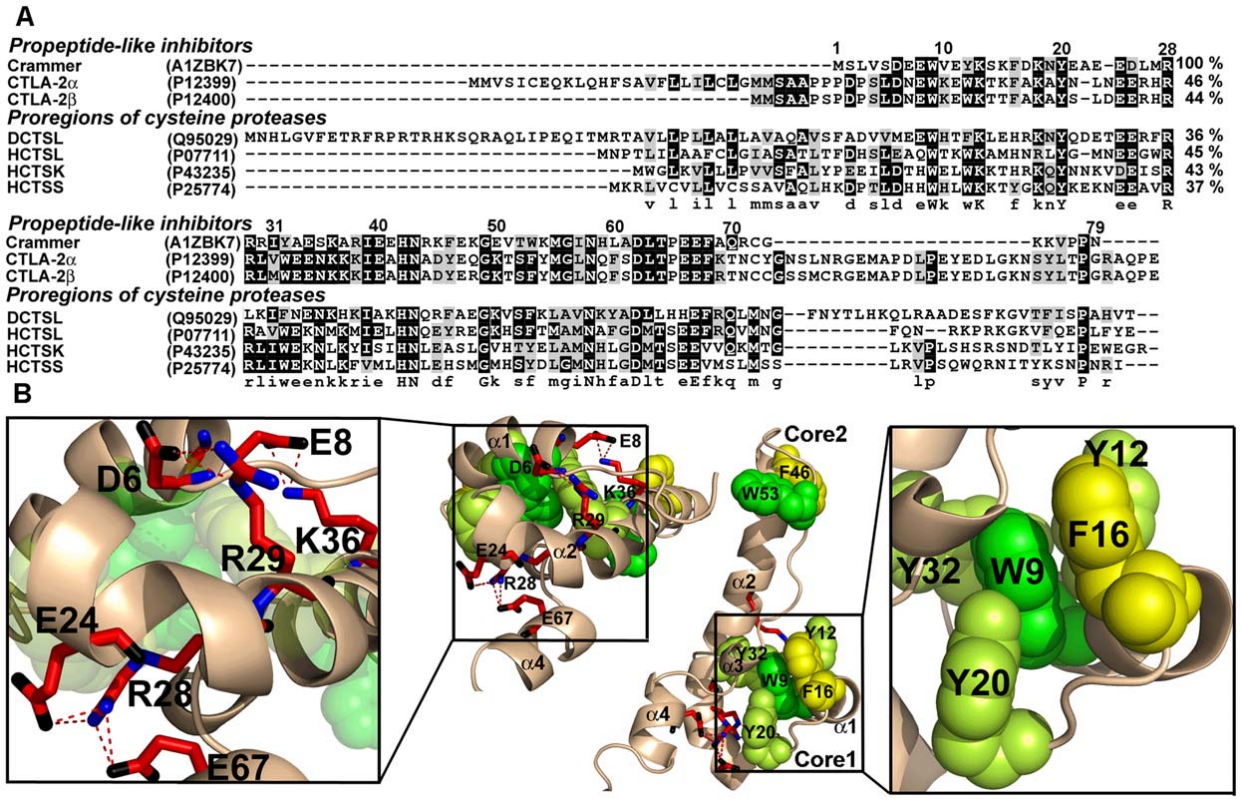
Sequence alignment and 3D structure of *Drosophila* crammer. (**A**) The multiple sequence alignment of crammer, propeptide-like cysteine protease inhibitors, and human cathepsin propeptides was conducted using the T-coffee algorithm [53] and the on-line server ExPASy (www.uniprot.org/). Accession numbers of individual sequences appear in parentheses. The identical, conserved, and semi-conserved residues are shaded in black, dark gray, and pale gray, respectively. In the consensus sequence, capital and small letters are used to indicate those residues found in all or most of the sequences, respectively. Residue numbering is based on that of the crammer sequence. DCTSL, propeptide of *Drosophila* cathepsin L. HCTSL, HCTSK, and HCTSS, propeptides of human cathepsin L, K, and S. (**B**) 3D structure of crammer shown as a ribbon diagram. The coordinates were taken from the Protein Data Bank (PDB) with the accession code of 2KTW [43]. The mutated residues in this study appear as spheres for the aromatic residues of hydrophobic Cores 1 and 2, and as sticks for the salt bridges. These images were prepared using PyMOL [54].

**Figure 2:**
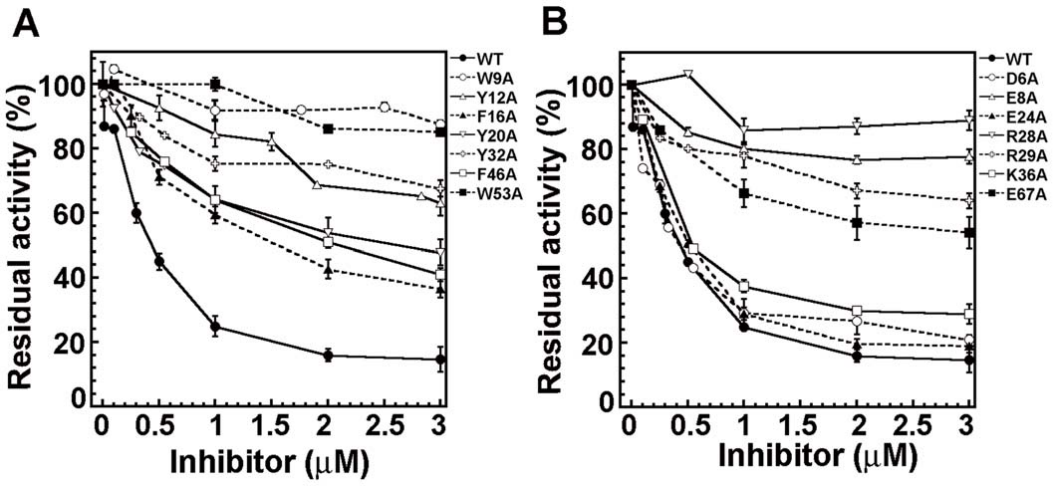
Inhibition of *Drosophila* CTSB by WT crammer and various mutants. The residual CTSB activity was determined at different concentrations of mutants by monitoring the release of AMC, which gives rise to fluorescence emission at 440 nm by excitation at 380 nm. (**A**) Comparison of the CTSB inhibition by WT crammer and the hydrophobic core mutants (**B**) CTSB inhibition by the salt bridge mutants.

Crammer contains seven aromatic residues in the hydrophobic Cores 1 and 2. These residues are located in the α-helix 1 (W9, Y12, F16), loop 1 (Y20), and the α-helix 2 (Y32) for hydrophobic Core 1, and the α-helix 2 (F46) and loop 2 (W53) for the hydrophobic Core 2 (Figure 1). Four salt bridges have been reported to connect the α-helices [43]. D6-R29 and E8-K36 connect the α-helices 1 and 2, while E24-R28 and R28-E67 connect the α-helices 2 and 4. Alanine scanning was used to elucidate the functional contributions of these residues in crammer (Figure 2, Tables 1 and 2). WT efficiently reduced CTSB activity by 85±4% at a concentration of 3 µM, whereas the F16A and F46A variants exhibit moderate inhibiting activities, reducing the activities of CTSB by 64±2% and 59±2%, respectively. On the contrary, the mutants Y12A, Y20A, and Y32A were less efficient against CTSB, and W9A and W53A were the least effective (Table 1). This study also investigates the functional importance of salt bridges (Figure 2B and Table 2). D6A, E24A, and K36A exhibit few changes in CTSB activity, whereas R29A and E67A moderately inhibit CTSB. On the other hand, E8A and R28A lost most of their inhibition activities. These results demonstrate that the aromatic residues (W9, Y12, Y20, Y32, and Y53) and the charge residues in the salt bridges (E8, R28, R29 and E67) are critical for CTSB inhibition.

**Table 1:** Activities and spectroscopic characteristics of the hydrophobic core mutants.

			CD			Intrinsic fluorescence					HSQC
Single	CTSB	C72S	Helical content (%)^b^		T_m_ (°C)		pH 4.0		pH 6.0		MG to OST^c^
mutants	activity^a^ %	Mutants	pH 4.0	pH 6.0	pH 6.0	*λ_max_* (nm)	*F_max_* (A.U.)	*λ_max_* (nm)	*F_max_* (A.U.)	*λ_max_* (pH 4–6) (nm)	
WT	14.7±3.7	C72S	32.9	54.0	58.0±0.6	336.5±0.5	1373.3	326.5±0.5	1527.4	10.0±0.0	O^d^
W9A	87.8±1.2	C72S/W9A	30.2	38.6	30.0±0.5	341.5±0.5	578.1	337.5±0.5	747.5	4.0±0.0	X^e^
Y12A	63.0±3.9	C72S/Y12A	32.6	46.1	33.0±0.6	338.5±0.5	1273.3	335.0±0.0	1584.5	3.5±0.5	X
F16A	34.6±2.4	C72S/F16A	31.7	44.2	42.6±0.7	345.5±0.5	1279.5	340.0±0.0	1660.6	5.5±0.5	P^f^
Y20A	47.8±3.8	C72S/Y20A	28.8	41.4	33.9±0.4	341.0±1.0	1444.3	338.5±0.5	1838.0	2.5±0.5	X
Y32A	67.6±2.5	C72S/Y32A	30.8	40.4	30.0±0.5	342.0±0.0	1326.6	338.0±0.0	1666.2	4.0±0.0	X
F46A	41.0±2.1	C72S/F46A	29.9	50.9	52.5±0.6	337.5±0.5	1569.3	326.5±0.5	1522.4	11.0±0.0	O
W53A	85.2±1.1	C72S/W53A	31.2	51.6	53.9±0.7	335.5±0.5	1593.7	325.5±0.5	1344.9	10.0±0.0	O

a *Drosophila* CTSB inhibition was measured in the presence of the mutants (3 µM).

b Experimental error for estimated helical content is ±3%.

c MG to OST, molten globule to ordered structure transition.

d O, the proteins enable to undergo the molten globule to ordered structure transition.

e X, the proteins are unable to undergo the molten globule to ordered structure transition.

f P, partial folding.

**Table 2:** Activities and spectroscopic characteristics of the salt bridge mutants.

			CD			Intrinsic fluorescence					HSQC
Single	CTSB	C72S	Helical content (%)^b^		T_m_ (°C)	pH 4.0		pH 6.0			MG to OST^c^
mutants	activity^a^ %	mutants	pH 4.0	pH 6.0	pH 6.0	*λ_max_* (nm)	*F_max_* (A.U.)	*λ_max_* (nm)	*F_max_* (A.U.)	*λ_max_* (pH 4–6) (nm)	
WT	14.7±3.7	C72S	32.9	54.0	58.0±0.6	336.5±0.5	1373.3	326.5±0.5	1527.4	10.0±0.0	O^d^
D6A	21.1±1.1	C72S/D6A	28.7	40.9	48.3±0.1	339.0±1.0	1431.8	329.5±0.5	1481.6	9.5±0.5	O
E8A	77.7±2.3	C72S/E8A	23.9	41.4	42.9±0.0	337.0±0.0	1529.3	333.0±0.0	1363.5	4.0±0.0	X^e^
E24A	19.0±2.1	C72S/E24A	28.5	40.5	46.6±0.4	337.5±0.5	1088.9	329.0±0.0	1300.4	8.5±0.5	O
R28A	89.0±3.1	C72S/R28A	22.2	43.5	42.5±0.4	336.0±0.0	1184.0	329.0±0.0	1382.0	7.0±0.0	P^f^
R29A	64.0±2.4	C72S/R29A	22.7	39.7	42.9±0.5	337.0±0.0	2131.0	333.0±0.0	1320.0	4.0±0.0	X
K36A	29.0±2.9	C72S/K36A	29.4	53.7	43.4±1.2	340.0±0.0	1007.0	332.0±0.0	1329.0	8.0±0.0	O
E67A	54.1±4.9	C72S/E67A	32.4	43.3	51.7±0.5	338.0±0.0	1415.0	327.5±0.5	1710.0	10.5±0.5	O

a *Drosophila* CTSB inhibition was measured in the presence of the mutants (3 µM).

b Experimental error for estimated helical content is ±3%.

c MG to OST, the molten globule to ordered structure transition.

d O, the proteins enable to undergo molten globule to ordered structure transition.

e X, the proteins are unable to undergo molten globule to ordered structure transition.

f P, partial folding.

### CD Spectroscopy of Crammer Alanine Mutants

#### Secondary structure content

**Figure 3:**
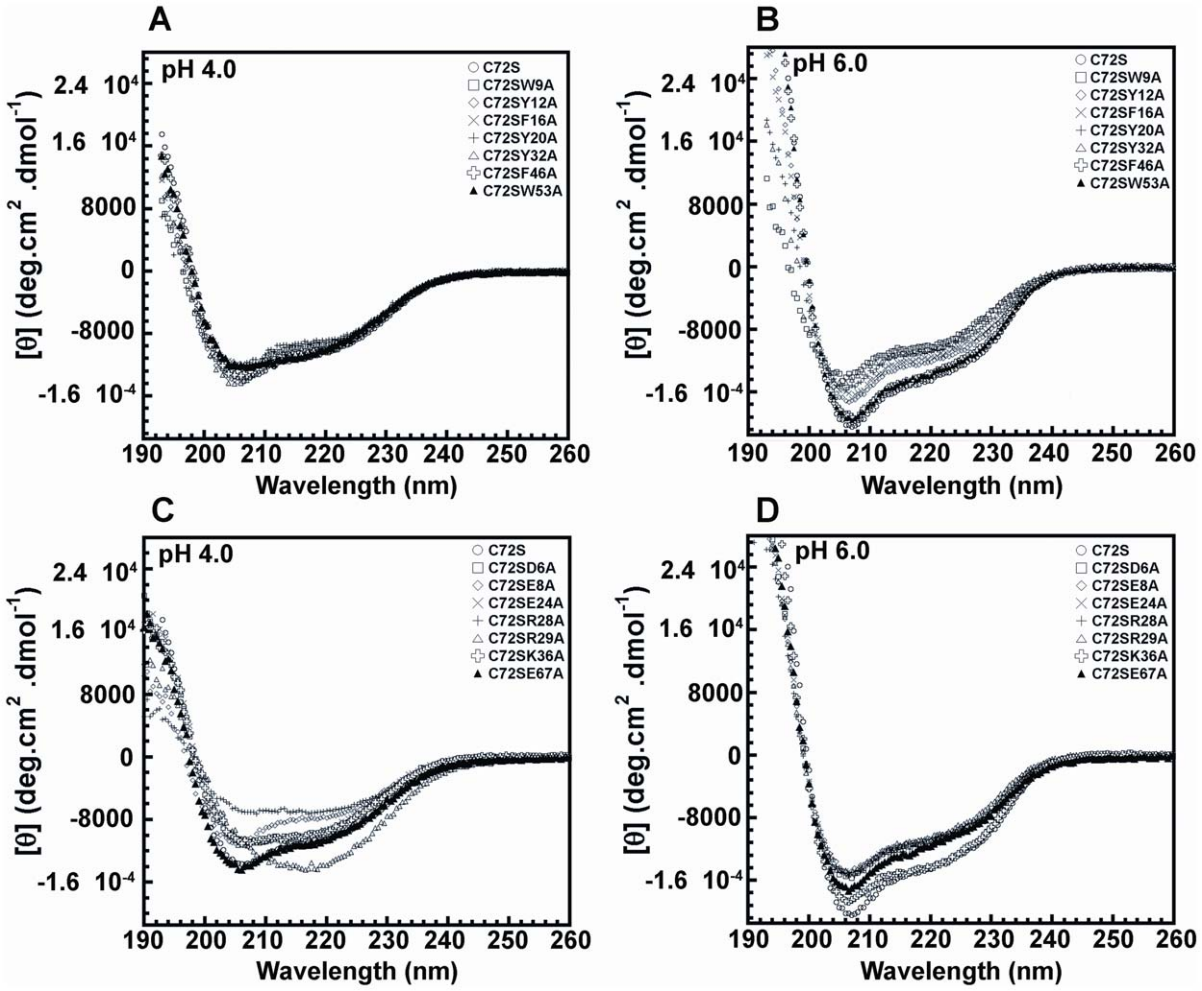
Far-UV CD spectra of C72S and C72S double mutants. CD spectroscopy was used to estimate the secondary structures of individual mutants. The CD signal in millidegrees was converted to mean-residue ellipticity [θ]. Far-UV CD spectra of (**A**) the hydrophobic core double mutants at pH4.0, and (**B**) at pH 6.0. (**C**) The salt bridge mutants at pH 4.0, and (**D**) at pH 6.0.

The WT crammer dimerizes *in vitro* through the formation of an intermolecular disulfide bond through the cysteine residue at position 72, Cys72. The replacement of Cys72 with Ser (i.e., C72S) completely abolishes the covalent dimer formation [28], [43]. C72S serves as a model system for investigating the pH-dependent structural properties of monomeric crammer [28]. At pH 4.0, all double mutants in the hydrophobic Core 1 exhibit nearly identical spectra with that of C72S, and all of them have similar helical contents (Figure 3, Tables 1 and 2). When the pH level increases to 6.0, C72S significantly increases the secondary structure content and presents a predominantly α-helical structure (Figure 3). However, at pH 6.0, the helical contents of C72S/W9A, C72S/Y12A, C72S/F16A, C72S/Y20A, and C72S/Y32A are significantly lower than that of C72S. This indicates that the alanine substitutions of these aromatic residues in the hydrophobic Core 1 disrupt the pH-dependent α-helical conformation of crammer. For the hydrophobic Core 2, C72S/F46A and C72S/W53A at pH 4.0 and 6.0 (Figures 3A and 3B) exhibit identical CD spectra with that of C72S. These data suggest that the alanine replacement at these positions does not cause significant perturbation to the protein structure. All salt bridge mutants (except for C72S/R29A and C72S/E67A) apparently reduce secondary structure contents at pH 4.0 (Table 2 and Table S4). The C72S/R29A mutant displays a distinctive spectral pattern compared to the other mutants (Figure 3C). Its secondary structure content is 22.7% α-helix and 31.4% β-strand, indicating that the β-strand conformation is dominant. At pH 6.0, however, these salt bridge mutants, with the exception of C72S/K36A, show significant loss of helical content (Figure 3D, Table 2 and Table S4). These results indicate that these charged residues are required to maintain the helical conformation of crammer.

#### Thermostability

**Figure 4:**
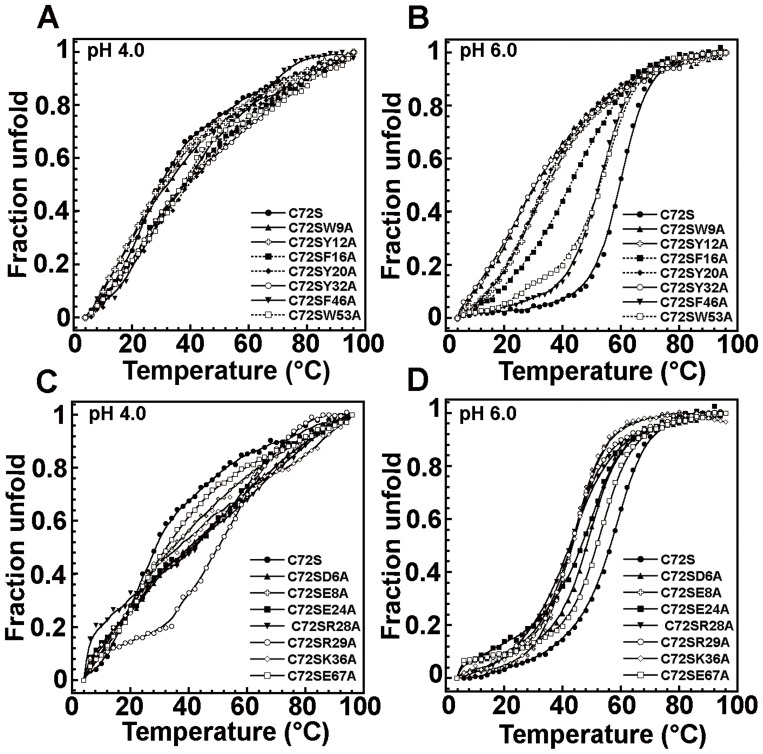
Thermal denaturation of C72S and C72S double mutants. Thermal denaturation was monitored at 208 nm from 4°C to 96°C. The unfolding curves of (**A**) the hydrophobic core double mutants at pH4.0, and (**B**) at pH 6.0. (**C**) The salt bridge mutants at pH 4.0, and (**D**) at pH 6.0.

Thermal denaturations of all crammer variants were monitored by CD spectroscopy at the wavelength of 208 nm. At pH 4.0, all double mutants in the hydrophobic Cores 1 and 2 displayed progressive and non-cooperative unfolding, which is consistent with that of C72S (Figure 4A). At pH 6.0, however, the denaturation curves of the mutants in the hydrophobic Core 1 are much less cooperative (Figure 4B) with the exception of C72S/F16A. This mutant exhibits a well-defined sigmoidal curve which corresponds to a two-state transition. The melting temperature, Tm (i.e., the midpoint of the unfolding transition) is significantly lower for all mutants in the hydrophobic Core 1 (Table 1). The hierarchy of thermal stability for these variants is in the order of C72S>C72S/F16A>C72S/Y20A>C72S/Y12A>C72S/Y32A>C72S/W9A. Our work also shows the free energy difference (ΔΔG_u_) and the population of denatured state at 20°C (Figure S1 and Tables 1 and S1). These mutants have negative ΔΔG_u_ values which that the mutation destabilizes the protein and increases the population of the denatured state. In contrast, C72S/F46A and C72S/W53A share denaturation curves similar to those of C72S at pH 6.0 (Figure 4B and Table 1). Hence, the removal of the aromatic side-chains at the hydrophobic Core 1, but not the hydrophobic Core 2, substantially decreases structural stability. We have also presented the thermal denaturation analysis for the salt bridge mutants at pH 4.0 and 6.0 (Figures 4C and 4D). At pH 4.0, the denaturation curves of all mutants are similar to the non-cooperative behavior of C72S. At pH 6.0, two double mutant cycles (C72S/D6A/R29A and C72S/R28A/E67A (Figure S8)) also exhibit non-cooperative curves (Figure S2), and their coupling energies (ΔΔG_int_) are 1.70 (D6-R29) and 2.77 (R28-E67) kcal/mol, respectively (Figure S3). Hence, these two salt bridges contribute significantly to crammer stability. The other salt bridge mutants exhibit sigmoidal and cooperative denaturation curves at pH 6.0; however, C72S/E8A, C72S/R28A, and C72S/R29A are less cooperative. All salt bridge mutants have lower Tm values (Table 2). The disruption of salt bridge residues particularly for E8, R28, and R29, reduces the thermostability of crammer.

### Intrinsic Fluorescence of the Crammer Mutants

**Figure 5:**
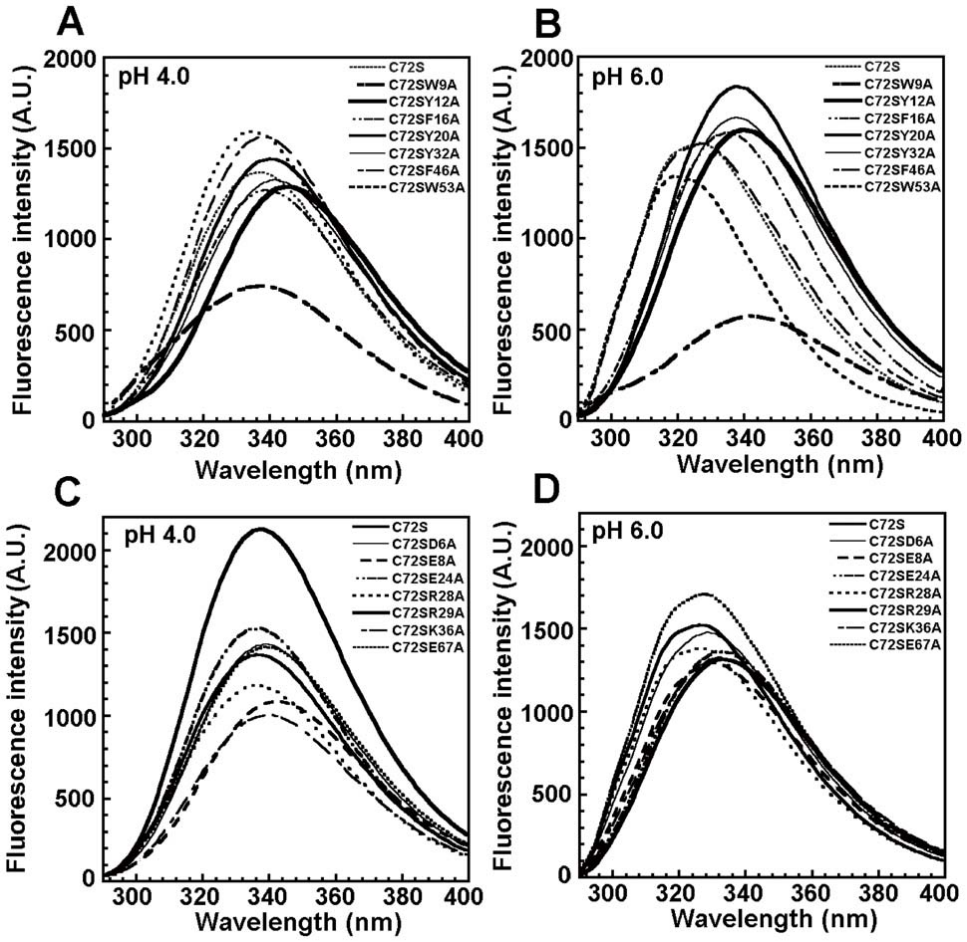
Intrinsic fluorescence of C72S and C72S double mutants. The fluorescence intensity of each mutant was recorded from 290 to 400 nm at an excitation wavelength of 280 nm at 25°C. Fluorescence emission spectra of (**A**) C72S and the hydrophobic core double mutants at pH 4.0, and (**B**) at pH 6.0. (**C**) The salt bridge mutants at pH 4.0, and (**D**) at pH 6.0. Tables 1 and 2 show the maximum fluorescence intensities and λ_max_.

This study uses intrinsic fluorescence to assess the effects of the mutations on the folding transition of crammer. At pH 4.0, C72S exhibits an emission maximum at 336.5 nm (Figure 5A), indicating that the tryptophan residues (W9 and W53) are exposed to solvent. At pH 6.0, C72S exhibits a blue-shift of 10 nm (λ_max = _326.5 nm), indicating that these residues become buried within the hydrophobic core of a folded state (Figure 5B). This blue-shifted maximum emission wavelength deviation (MEWD) is accompanied by a 154 (A.U.) increase of relative fluorescence intensity. A similar experiment has been applied to other crammer variants (Figures 5A and 5B). Crammer has two tryptophan residues in its primary sequence: W9 and W53. Therefore, W53 is the only fluorescence probe for tryptophan fluorescence for C72S/W9A; likewise, W9 is the sole fluorescence probe for C72S/W53A. At pH 4.0, C72S/W9A exhibits an emission λ_max_ at 342 nm, and at pH 6.0, the emission λ_max_ is at 338 nm (i.e., a blue-shift of 4 nm). For C72S/W53A, a blue-shift of its λ_max_ value is from 336 nm to 326 nm when the pH increases from 4.0 to 6.0. The solution structure of C72S shows that W9 is buried in the hydrophobic Core 1 (relative solvent accessible surface area, SASA, 0.2%), whereas W53 is more accessible to solvent (SASA, 37.7%). The replacement of W9 with alanine causes greater disruption of the structural elements and stability which are associated with the pH-dependent disorder-to-order transition.

Other mutants in the hydrophobic Core 1 (C72S/Y12A, C72S/F16A, C72S/Y20A and C72S/Y32A) have lower MEWD values (5–8 nm) (Table 1). These data indicate that the removal of any of the aromatic side-chains in the hydrophobic Core 1 disrupts hydrophobic interactions, thereby disturbing the disorder-to-order transition in crammer. On the contrary, C72S/F46A and C72S/W53A show pH-dependent blue-shifts (10 nm) which is similar to that of WT crammer. This indicates that the packing of the aromatic resides at the hydrophobic Core 2 is less critical to protein folding. For the salt bridge mutants, C72S/D6A, C72S/E24A, and C72S/E67A have blue-shifts which are similar to those of C72S (Figures 5C and 5D, Table 2); however, C72S/E8A, C72S/R28A, C72S/R29A, and double mutant cycles (C72S/D6A/R29A and C72S/R28A/E67A) apparently reduce MEWD values (Table 2). Therefore, E8, R28, R29, and two salt bridges (D6-R29 and R28-E67) are important for the folding transition of crammer.

### ^1^H-^15^N HSQC NMR Spectroscopy

**Figure 6:**
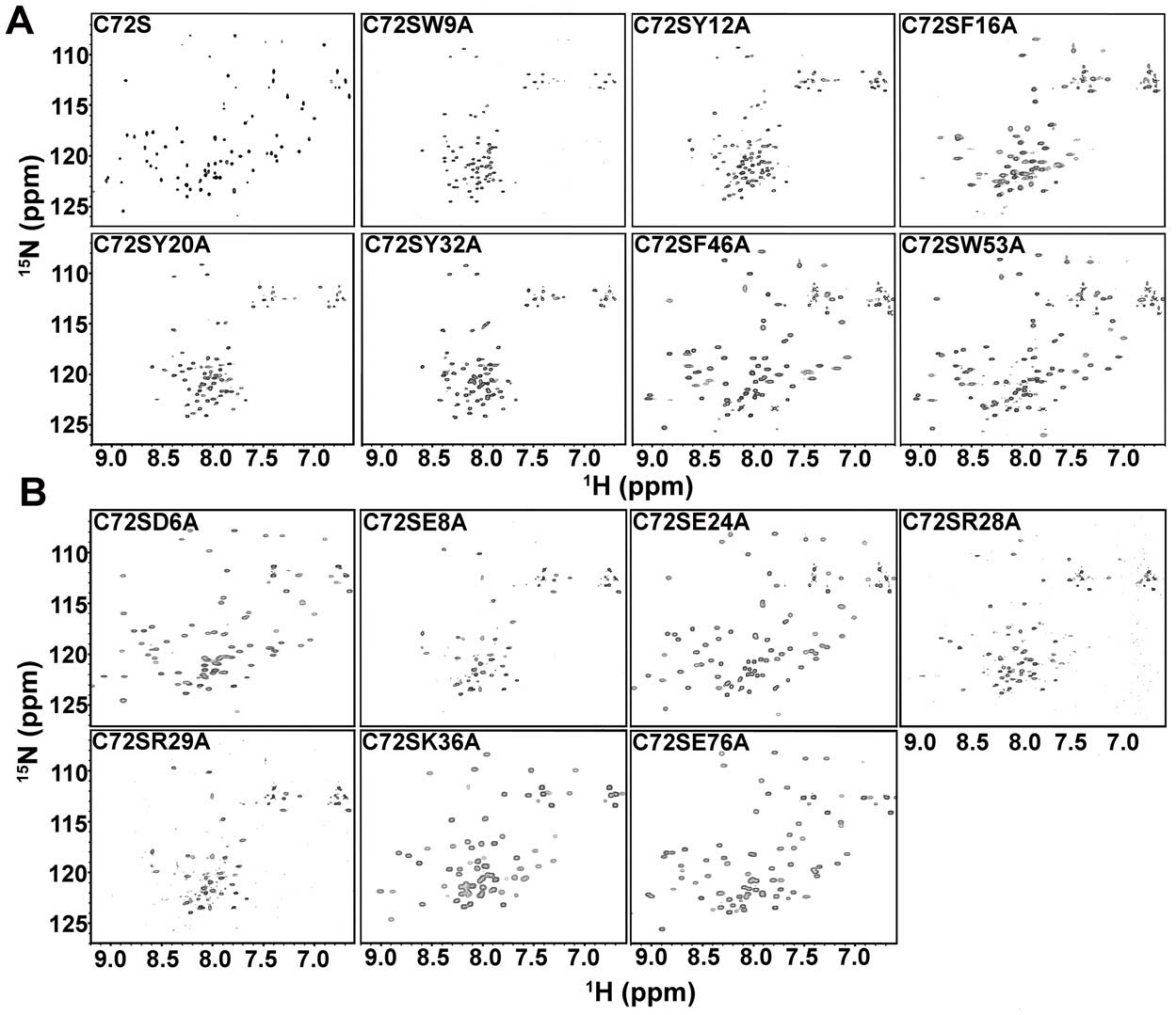
^1^H-^15^N-HSQC spectra of C72S and C72S double mutants. 2D ^1^H-^15^N-HSQC NMR spectra of (**A**) C72S and the hydrophobic core double mutants at pH 6.0, and (**B**) the salt bridge mutants at pH 6.0.

Monomeric crammer is a molten globule at pH 4.0, but adopts an ordered structure when bound to CTSB. The well-dispersed cross-peaks from the ^1^H-^15^N HSQC spectrum (most of which can be superimposed on those observed for C72S at pH 6.0) [28], provide evidence for this transition. Based on these findings, this work investigates the molten globule-to-ordered structure transition of all C72S double mutants by comparing their ^1^H-^15^N HSQC spectra at pH 4.0 and at pH 6.0. Figure S4 shows that C72S displays poorly dispersed cross-peaks at pH 4.0, corresponding to a molten globule state. Similar phenomena are observed for other mutants at the hydrophobic Cores 1 and 2. A new set of well-dispersed cross-peaks of C72S emerges at pH 6.0; however, this indicates the formation of a well-folded tertiary structure (Figure 6A). Most of the double mutants at pH 6.0 still remains poorly dispersed, except for C72S/F16A, C72S/F46A, and C72S/W53A. C72S/F16A shows fewer dispersed cross peaks when compared to C72S. This information indicates a partially folded conformation for this mutant. On the other hand, C72S/F46A and C72S/W53A exhibit well-dispersed amide proton chemical shifts (Figure 6A) and their observed cross-peaks resemble those of C72S at pH 6.0. The aromatic residues at the hydrophobic Core 1, but not the hydrophobic Core 2, are therefore crucial for maintaining the molten globule-to-ordered structure transition in crammer. These results are in good agreement with the thermostability and intrinsic fluorescence data. The salt bridge mutants, C72S/D6A, C72S/E24A, C72S/K36A, and C72S/E67A display well-dispersed cross-peaks in the ^1^H-^15^N HSQC spectra at pH 6.0 (Figure 6B), which signifies the presence of the well-folded structures and the disorder-to-order transition. Figure 6B also show that C72S/R28A behaves similarly to C72S/F16A since both exhibit a partially folded conformation at pH 6.0 while for C72S/E8A and C72S/R29A show poorly dispersed cross-peaks, which suggest a molten globule-like state. Taken together, these results show that E8, R28, and R29 play important roles in maintaining the folding transition in crammer.

## Discussion

Using alanine substitutions, this study systematically investigates the importance of the aromatic residues in crammer. We found that W9, Y12, F16, Y20, and Y32 in the hydrophobic Core 1 make important contributions to folding transition and CTSB inhibition. Mutations on these residues in crammer substantially altered the proper conformation of α1 and α2-helices, significantly decreased the structural stability, and increased the population of the denatured state at 20°C (Figure S1). This helical conformation serves as a nucleus for the formation of the ordered tertiary structure of crammer at pH 6.0. The disruption of the molten globule-to-ordered structure transition in crammer reduces the inhibition potency against CTSB. The activation of human cathepsin requires the cleavage of its proregion under acidic condition [44]. The cleaved proregion exists as a molten globule [45] with poor inhibitory activity against cathepsin [46]. Unlike the proregion of human cathepsin, the propeptide-like crammer is a strong inhibitor against cathepsin under acidic conditions and the tight binding of crammer is associated with a molten globule-to-ordered structure transition [28]. The disruption of this structure transition (such as in the case of the W9A mutant) causes poor inhibitory ability and triggers W9A proteolysis through CTSB (Figure S5). These results suggest the importance of folding coupled binding (i.e., induced fit) and inhibition. The inefficient inhibition of the cleaved disordered proregion against cathepsin may result from defects of pH- or target binding-dependent disordered-to-ordered transition. This in turn renders the propeptides more susceptible to cathepsin proteolysis.

Crammer has a high sequence homology (>36%) with the propeptides of cysteine proteases (Figure 1), and its hydrophobic Core 1 contains five conserved aromatic residues (W9, Y12, F16, Y20, and Y32). The first and the fourth residues for all propeptide-like inhibitors and propeptides are tryptophan and tyrosine, respectively (Figure 1A). Therefore, this study proposes a conserved aromatic motif, WX_2_(F/Y/W)X_3_(F/Y)X_3_YX_12_(F/Y/W) (Figure 1A) that is essential for the formation of the hydrophobic Core 1. More importantly, this study presents an analysis of the π–π interactions among the aromatic residues of the hydrophobic cores in crammer and the propeptides of human cathepsins L, K, and S using the Protein Interactions Calculator (PIC) [47]. The first aromatic residue of crammer, W9, is located at the center of hydrophobic Core 1, and its bulky indole ring serves as an anchor for interacting with other aromatic residues through π–πinteractions ( and Table S2). In contrast, the corresponding entity in the propeptides of human cathepsins L, K, and S is the second tryptophan residue. Although these propeptides have different arrangements and orientations at this unique tryptophan, they also share π–πinteractions similar to those of crammer to promote the formation of a hydrophobic core. This tryptophan residue also exhibits van der Waals interactions with the surrounding charged or hydrophobic residues in the hydrophobic core (Table S3). Therefore, mutation at any of the aromatic residues in the hydrophobic Core 1 of crammer can destroy these interactions and the core formation. Consequently, this would lead to the disruption of the molten globule-to-ordered structure transition.

Crammer has two tryptophan residues in its primary sequence (W9 and W53), but these two residues have different effects on the Trp fluorescence emission. C72S/W53A apparently exhibits a larger blue-shift than C72S/W9A (Table 1). This suggests that C72S/W9A displays a higher degrees of solvent exposure of the Trp side chains in the structure of crammer. Moreover, C72S/W9A has lower protein stability and higher population of the denatured state (Figure S1 and Table S1). Hence, we propose that the replacement of W9 with alanine causes greater disruption of π–π interactions, thereby significantly disturbing the protein stability and structure folding, as well as the disorder-to-order transition in crammer. In contrast, C72S/W53A maintains the similar structure and stability with that of C72S, indicating that the packing of W53 at the hydrophobic Core 2 is less critical to protein folding. However, W53A also remarkably decreased CTSB inhibition, indicating that the loss of function is not because of folding deficiency. The exposed indole ring of W53 may be involved in CTSB binding. Given the sequence similarity (Figure 1), crammer and the propeptides seem to share similar binding modes. The complex structure of procathepsin S shows that the propeptide side chain of W58 is in contact with the side chain of W153 of the pro-segment binding loop (PBL) [48]. In procathepsin L, F56 of the propeptide makes close contacts with Y151 of the PBL [49] (Figure S7). This study uses homology modeling to generate the structure of *Drosophila* procathepsin B to clarify the role of W53 in crammer. This model was superimposed to the structures of procathepsins S (CTSS), K (CTSK), and crammer (Figure S7). Results show that the indole ring of W53 in crammer adopts a similar orientation as the propeptides and may interact with Y261 of the PBL of *Drosophila* CTSB. In rat cathepsin B propeptide, alanine substitution at this residue also apparently reduces cathepsin inhibition [50].

This study also investigates the importance of the salt bridges in crammer. The proposed double mutant cycle analysis shows that D6-R29 and R28-E67 are critical for crammer stability (Figure S3). The replacement of the charged residues E8, R28, R29, and E67 with alanine considerably reduces their CTSB inhibition activity, whereas the replacement of D6, E24, and K36 does not. C72S/E8A and C72S/R29A lose the disorder-to-order transition, thus reducing their inhibitory activity. Likewise, C72S/R28A only has the partial folded structure at pH 6.0 and, therefore exhibits lower cathepsin inhibition. Although E8 and R29 form salt bridges with K36 and D6, respectively, only the former two residues contribute significantly to protein folding and CTSB inhibition. These asymmetric contributions can be rationalized in terms of the relative positions of individual residues in the 3D structure. D6 is located in loop 1, E8 in α-helix 1, E24 in loop 2, R28 and R29 in α-helix 2, K36 in the middle of α-helix 2, distant from the hydrophobic Core 1, and E67 is located in α-helix 4. Because α-helices 1 and 2 form the backbone of the hydrophobic Core 1, mutations at these two helices are likely to have greater effect on core formation. C72S/E67A exhibits thermostability and structure transition results similar to those of C72S, but its CTSB inhibition activity is poor. Multiple sequence alignment (Figure 1A) shows that this glutamic acid is highly conserved among crammer, CTLA-2α, CTLA-2β and propeptides of human cathepsins L, K, and S. The salt bridge between R28 and E67 of crammer is structurally equivalent to the salt bridges R31-E70, R3-E70, and R33-E72 of the propeptides of human cathepsins L [49], K [51], [52], and S [48], respectively. The R33-E72 salt bridge in human cathepsin S contributes to the proper orientation of the α-helix toward the active site cleft [48]. Therefore, this study proposes that the R28-E67 salt bridge, connecting α-helices 2 and 4 in crammer, is essential for the proper orientation of α-helix 4 with respect to the active site of CTSB. Therefore, mutation on E67 may cause misalignment of α-helix 4, leading to a weaker binding affinity and lower CTSB inhibition activity.

### Conclusion

This study uses alanine scanning to identify the critical amino acids of *Drosophila* crammer in terms of CTSB inhibition, structure, stability, and disorder-to-order transition. The results of this study show that the conserved aromatic residues W9, Y12, Y20, and Y32 at the hydrophobic Core 1 are critical for folding transition and CTSB inhibition. These residues form π-π interactions and van der Waals contacts to maintain the integrity of the hydrophobic core. This in turn promotes the pH-dependent molten globule-to-ordered structure transition in crammer. The propeptides of human cathepsins adopt different steric arrangements at these corresponding residues, but they still adopt similar π-π stacking interactions to stabilize the hydrophobic core and the overall folding. Hence, this π-π stacking is required for the propeptides and crammer to trigger efficient inhibition activity against cysteine protease. The solvent-exposed W53 at the hydrophobic Core 2 of crammer probably directly interacts with PBL of *Drosophila* CTSB to express its CTSB inhibition. Finally, the conserved charged residues in crammer provide an interaction network to promote the helix formation and maintain thermostability. Specifically, the disruption of the R28-E67 salt bridge in crammer apparently disturbs the structure transition, thus reducing CTSB inhibition. These results provide an insight into the structural and functional roles of key amino acids in crammer, which is useful for the development of cysteine protease inhibitors.

## Supporting Information

Figure S1 **Comparison of protein structure and stability of C72S and all double mutants at pH 6.0.** To clearly realize the relationship of protein structure and stability upon the mutation, we made a figure to compare the ellipticity (**A**), the MEWD value (**B**), the unfolding free energy (ΔG_u_) (**C**), and the fraction unfold (**D**) at 20°C. The CD signal of each mutant was recorded at 208 nm and presented in millidegree. The MEWD values were obtained from the results of the intrinsic fluorescence measurement. The fraction unfold and the unfolding free energy were determined from the thermal denatureation curves [41]. At 4°C, the mutants at the hydrophobic core 1 apparently have the lower ellipticity, the MEWD value, and ΔG_u_ as compared with those of C72S (Tables 1 and S1). As increased the temperature to 20°C, protein structure and stability of these mutants are changed at the same time. This result leads to increase the population of the denatured state. As for other mutants in hydrophobic core 2, they share the similar structure and stability with that of C72S. Thus, they have similar the population of the denatured state. Finally, upon the mutation, these proteins have different impact on the protein structure and stability, thus resulting in a variety of population of the denatured state at 20°C and at pH 4.0 and 6.0.
(TIF)

Figure S2 **Thermal denaturation of C72S and salt bridge mutants.** Thermal unfolding curves of C72S, double mutants (C72S/D6A, C72S/R29A, C72S/R28A, and C72S/E67A) and triple mutants (C72S/D6A/R29A and C72S/R28A/E67A) were monitored at 208 nm from 4°C to 96°C at pH 6.0.
(TIF)

Figure S3 **Coupling energy of salt bridge (ΔΔG_int_) at pH6.0. (A)** To understand contribution of the salt bridges in protein stability, the double-mutant cycle analysis is employed [55]–[57]. (**A**) The scheme shows that the pair-wise interaction energy (ΔΔG_int_) is calculated from the unfolding free energy (ΔG_u_) of wild-type (WT) protein, single-mutants (M^+ve^ and M^−ve^), and double-mutant (DM). The substitutions are indicated inside the boxes and the ΔΔG_u_ values for processes A–D are shown along the arrows. The ΔΔG_u_ value is the difference of the unfolding free energies due to mutation, The ΔΔG_int_ value is then calculated using an equation that is showed in the figure. The circles, labeled with **“−”, “+”,** and blank signs mean a negative charged residue, a positive charged residue and an alanine substitution, respectively. (**B**) The coupling energy (ΔΔG_int_) for salt bridge, D6-R29, is 2.77 kcal mol^−1^. (**C)** ΔΔG_int_ for salt bridge, R28-E67, is 1.70 kcal mol^−1^. The positive ΔΔG_int_ indicates that these two salt bridges have significantly contribution to the stability of crammer.
(TIF)

Figure S4 **^1^H-^15^N-HSQC spectra of the hydrophobic core 1 double mutants of crammer at pH 4.0.**
(TIF)

Figure S5 **Digestion of crammer single mutants by CTSB.** In order to evaluate the proteolysis resistance, 3 µM of each single mutant (W9A, F16A, R28A, and C72S) and wild-type crammer (Cer) were incubated with *Drosophila* cathepsin B (CTSB, 100 nM) in 100 mM sodium acetate (pH 5.0), 1 mM EDTA and 2 mM DTT at 25°C for 1 and 2 hours. The digested protein solutions were further analyzed by 13% (w/v) Tricine-SDS/PAGE. The molecular weight of single mutants is ∼9.5 kD. Cer and C72S are resistant to CTSB digestion, but, however, W9A, F16A, and R28A exhibited onset of digestion after incubated with CTSB for 1 and 2 hours.
(TIF)

Figure S6 **Structural alignment of atoms of crammer with the propeptides of three human cathepsins.** Superimposition of the Cα atoms of crammer (red; PDB entry 2KTW) with those of the human cathepsin propeptides L (light grey; PDB entry 1CS8 [49], [58]), K (dark grey; PDB entry 1BY8 [52]) and S (black; PDB entry 2C0Y [48]) yields a moderate pair-wise positional root mean square deviation (RMSD) of 4.1 Å, 5.6 Å and 4.4 Å, respectively. The relatively large positional deviation is mainly due to the different orientations of the individual-helices. (**A**) Orientation of the aromatic residues in the hydrophobic core 1 of crammer. (**B**) Superposition of the conserved aromatic residues of the propeptides of human cathepsin L, K and S with those of crammer. (**C**) Orientations of the aromatic residues in the hydrophobic cores of the propeptides of human cathepsins L, K, and S. The picture was prepared with PyMOL [54].
(TIF)

Figure S7 **Structural alignment of crammer, the human procathepsins K and S, and the modeled structure of** ***Drosophila*** **procathepsin B.** 3D coordinates for crammer (yellow), and human procathepsins K (blue) and S (red), are taken from the PDB (entries 2KTW [43], 1BY [52], and 2C0Y [48], respectively.) The *Drosophila* procathepsin B (light grey) structure is modeled using Modeller [59]–[62], based on the structure of human procathepsin B (PDB code: 3PBH [63]). The stereochemical quality of the model was examined using Procheck [64], [65]. In addition to the results of superposition of human procathepsins K and S with respect to crammer in Figure S2, the positional Cα RMSD between the modeled *Drosophila* procathepsin B and crammer is 10.2 Å. Insert: Expanded view of the interactions between the conserved aromatic residues of the propeptides and the prosegment binding loop (PBL) of mature cathepsin: W53 of crammer and W27 of the propeptide of *Drosophila* procathepsin B interact with W261 of PBL of mature *Drosophila* CTSB. Additionally, Y58 of human procathepsin K, and Y56 of procathepsin S make contacts with the aromatic residues of the PBL of mature human cathepsin K at Y150, andcathepsin S at Y153. The picture was prepared with PyMOL [5].
(TIF)

Figure S8 **Expression profiles of double mutants cycles (C72S/D6A/R29A, C72S/E8A/K36A and C72S/R28A/E67A).** The protein samples were analyzed by 13% (w/v) Tricine-SDS/PAGE. An arrow indicates the expected position of the proteins. M, protein molecular weight standard; C, uninduced control without IPTG induction; I, expression of the double mutant cycles after IPTG induction.
(TIF)

Table S1 **Thermal dynamic parameters of mutant proteins at pH 6.0.** The entropy (ΔS) and the enthalpy (ΔH) of mutants are determined from the thermal denaturation curves [41]. After that, the unfolding free energy (ΔG_u_) at 277K and 293 K can be deduced according to Gibbs free energy equation.
(DOCX)

Table S2 **Analysis of the π-π interactions of crammer and the propeptides of human cathepsins L (HCTSL), K (HCTSK) and S (HCTSS).**
(DOCX)

Table S3 **Hydrophobic contact analysis.** The software, Ligplot, [66] was used to analyze the hydrophobic contacts for five conserved aromatic residues (W9, Y12, F16, Y20, and Y32). These residues play an important role in the stabilization of the hydrophobic core 1 of crammer.
(DOCX)

Table S4 **The predicted helical propensities of double mutants in salt bridges by the agadir program (** http://agadir.crg.es **).**
(DOCX)
