# Supplementary figures and images for: Residue-Specific Annotation of Disorder-to-Order Transition and Cathepsin Inhibition of a Propeptide-Like Crammer from D. melanogaster
Source: PLoS One. 2013 Jan 21;8(1):e54187. doi: 10.1371/journal.pone.0054187 (PMC3551606; doi:10.1371/journal.pone.0054187)

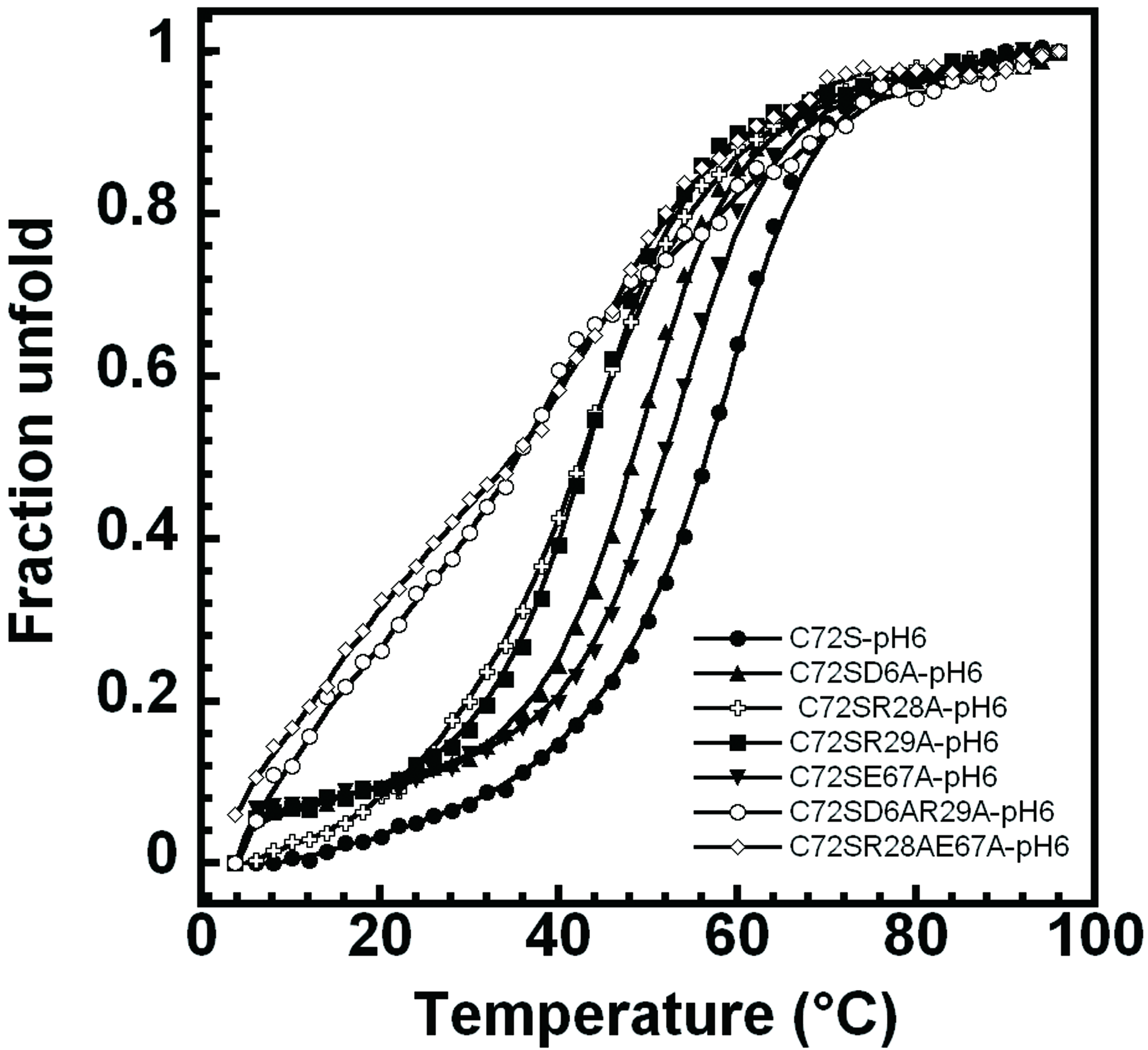

Supplement: Figure S2 — Thermal denaturation of C72S and salt bridge mutants. Thermal unfolding curves of C72S, double mutants (C72S/D6A, C72S/R29A, C72S/R28A, and C72S/E67A) and triple mutants (C72S/D6A/R29A and C72S/R28A/E67A) were monitored at 208 nm from 4°C to 96°C at pH 6.0. (TIF) [file pone.0054187.s002.tif]

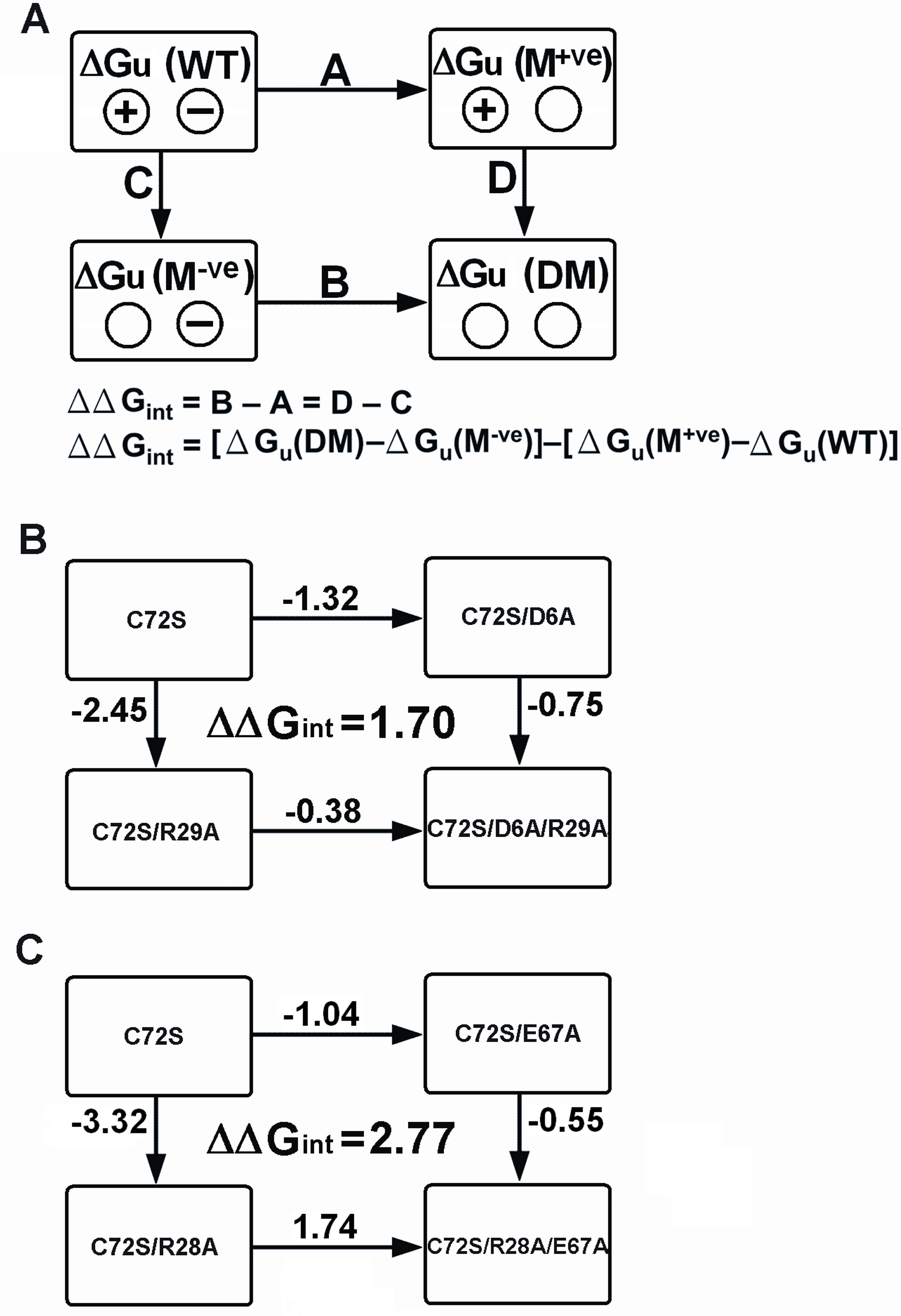

Supplement: Figure S3 — Coupling energy of salt bridge (ΔΔGint) at pH6.0. (A) To understand contribution of the salt bridges in protein stability, the double-mutant cycle analysis is employed [55]–[57]. (A) The scheme shows that the pair-wise interaction energy (ΔΔGint) is calculated from the unfolding free energy (ΔGu) of wild-type (WT) protein, single-mutants (M+ve and M−ve), and double-mutant (DM). The substitutions are indicated inside the boxes and the ΔΔGu values for processes A–D are shown along the arrows. The ΔΔGu value is the difference of the unfolding free energies due to mutation, The ΔΔGint value is then calculated using an equation that is showed in the figure. The circles, labeled with “−”, “+”, and blank signs mean a negative charged residue, a positive charged residue and an alanine substitution, respectively. (B) The coupling energy (ΔΔGint) for salt bridge, D6-R29, is 2.77 kcal mol−1. (C) ΔΔGint for salt bridge, R28-E67, is 1.70 kcal mol−1. The positive ΔΔGint indicates that these two salt bridges have significantly contribution to the stability of crammer. (TIF) [file pone.0054187.s003.tif]

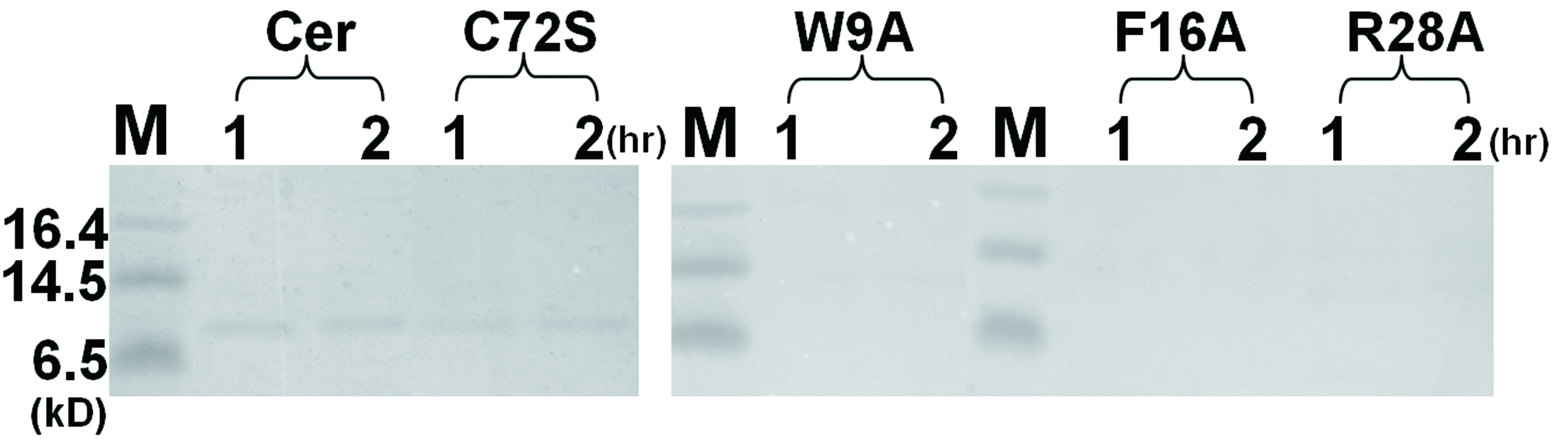

Supplement: Figure S5 — Digestion of crammer single mutants by CTSB. In order to evaluate the proteolysis resistance, 3 µM of each single mutant (W9A, F16A, R28A, and C72S) and wild-type crammer (Cer) were incubated with Drosophila cathepsin B (CTSB, 100 nM) in 100 mM sodium acetate (pH 5.0), 1 mM EDTA and 2 mM DTT at 25°C for 1 and 2 hours. The digested protein solutions were further analyzed by 13% (w/v) Tricine-SDS/PAGE. The molecular weight of single mutants is ∼9.5 kD. Cer and C72S are resistant to CTSB digestion, but, however, W9A, F16A, and R28A exhibited onset of digestion after incubated with CTSB for 1 and 2 hours. (TIF) [file pone.0054187.s005.tif]

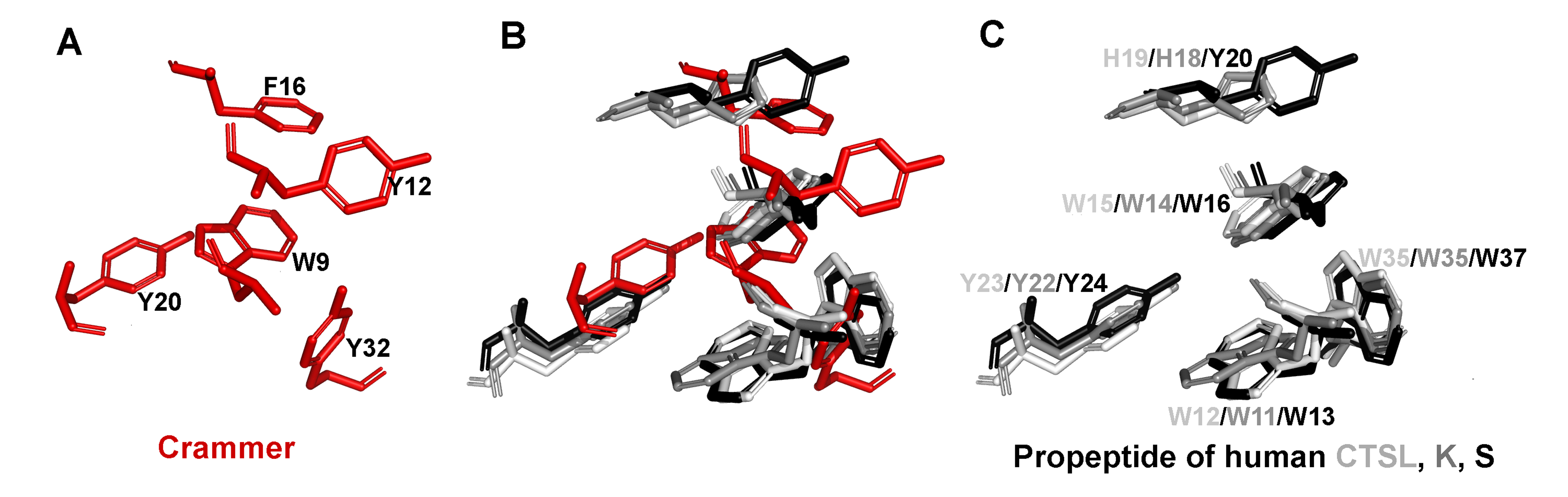

Supplement: Figure S6 — Structural alignment of atoms of crammer with the propeptides of three human cathepsins. Superimposition of the Cα atoms of crammer (red; PDB entry 2KTW) with those of the human cathepsin propeptides L (light grey; PDB entry 1CS8 [49], [58]), K (dark grey; PDB entry 1BY8 [52]) and S (black; PDB entry 2C0Y [48]) yields a moderate pair-wise positional root mean square deviation (RMSD) of 4.1 Å, 5.6 Å and 4.4 Å, respectively. The relatively large positional deviation is mainly due to the different orientations of the individual-helices. (A) Orientation of the aromatic residues in the hydrophobic core 1 of crammer. (B) Superposition of the conserved aromatic residues of the propeptides of human cathepsin L, K and S with those of crammer. (C) Orientations of the aromatic residues in the hydrophobic cores of the propeptides of human cathepsins L, K, and S. The picture was prepared with PyMOL [54]. (TIF) [file pone.0054187.s006.tif]

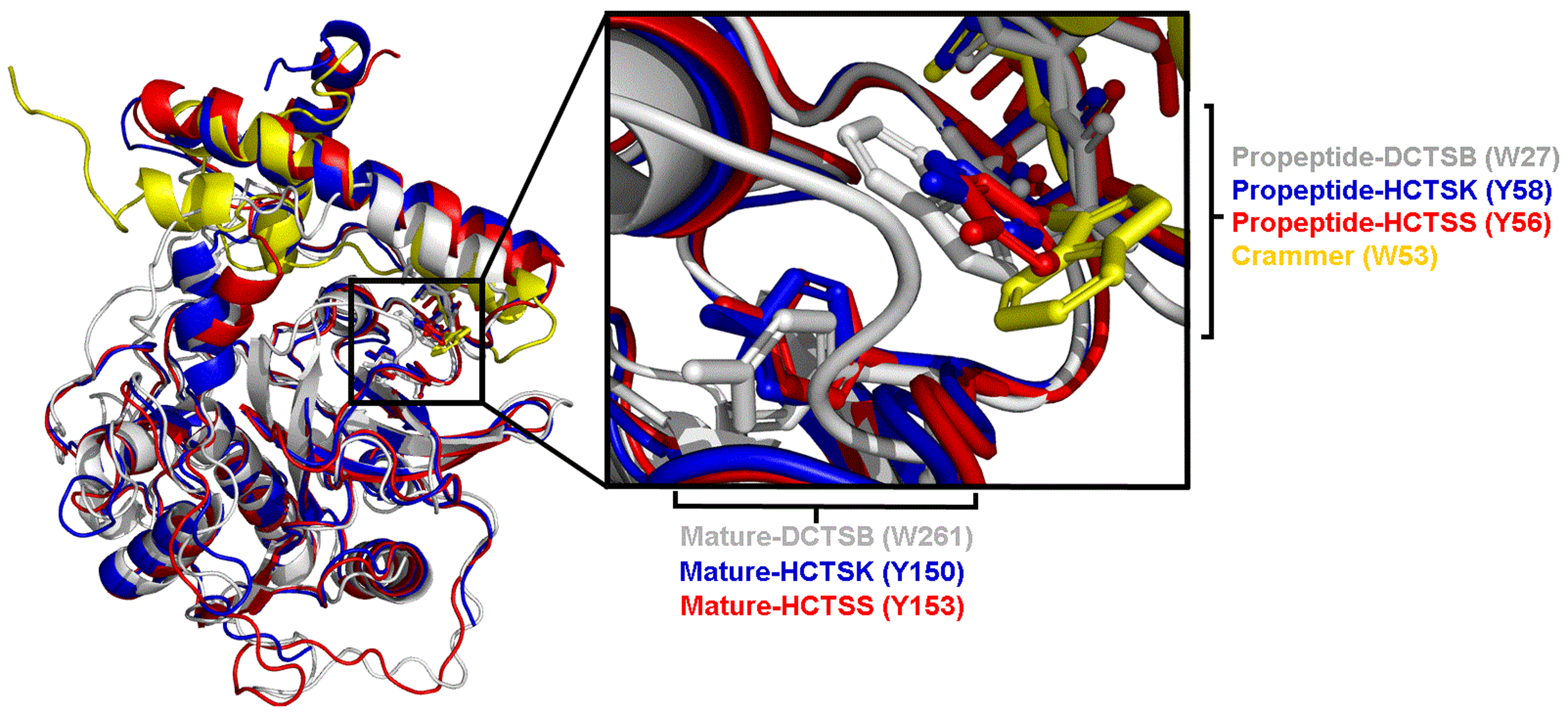

Supplement: Figure S7 — Structural alignment of crammer, the human procathepsins K and S, and the modeled structure of Drosophila procathepsin B. 3D coordinates for crammer (yellow), and human procathepsins K (blue) and S (red), are taken from the PDB (entries 2KTW [43], 1BY [52], and 2C0Y [48], respectively.) The Drosophila procathepsin B (light grey) structure is modeled using Modeller [59]–[62], based on the structure of human procathepsin B (PDB code: 3PBH [63]). The stereochemical quality of the model was examined using Procheck [64], [65]. In addition to the results of superposition of human procathepsins K and S with respect to crammer in Figure S2, the positional Cα RMSD between the modeled Drosophila procathepsin B and crammer is 10.2 Å. Insert: Expanded view of the interactions between the conserved aromatic residues of the propeptides and the prosegment binding loop (PBL) of mature cathepsin: W53 of crammer and W27 of the propeptide of Drosophila procathepsin B interact with W261 of PBL of mature Drosophila CTSB. Additionally, Y58 of human procathepsin K, and Y56 of procathepsin S make contacts with the aromatic residues of the PBL of mature human cathepsin K at Y150, andcathepsin S at Y153. The picture was prepared with PyMOL [5]. (TIF) [file pone.0054187.s007.tif]
