# Supplementary material for: Residue-Specific Annotation of Disorder-to-Order Transition and Cathepsin Inhibition of a Propeptide-Like Crammer from D. melanogaster
Source: PLoS One. 2013 Jan 21;8(1):e54187. doi: 10.1371/journal.pone.0054187 (PMC3551606; doi:10.1371/journal.pone.0054187)
Supplement: Table S1 — Thermal dynamic parameters of mutant proteins at pH 6.0. The entropy (ΔS) and the enthalpy (ΔH) of mutants are determined from the thermal denaturation curves [41]. After that, the unfolding free energy (ΔGu) at 277K and 293 K can be deduced according to Gibbs free energy equation. (DOCX) [file pone.0054187.s009.docx]

**Table S1. Thermal dynamic parameters of mutant proteins at pH 6.0. The entropy (ΔS) and the enthalpy (ΔH) of mutants are determined from the thermal denaturation curves [**[**1**](#_ENREF_1)**]. After that, the unfolding free energy (ΔG_u_) at 277K and 293 K can be deduced according to Gibbs free energy equation.**

|  |  |  | **ΔG_u_ (kcal mol^-1^)** | | **ΔΔG_u (mutant － C72S)_ (kcal mol^-1^)^a^** | | |
| --- | --- | --- | --- | --- | --- | --- | --- |
| **Protein** | **ΔS (cal mol^-1^ K^-1^)** | **ΔH (kcal mol^-1^)** | **277 K** | **293 K** | | **277 K** | **293 K** |
| C72S | 149.00 | 49.62 | 8.32 | 5.94 | 0 | | 0 |
| C72S/W9A | 69.46 | 22.19 | 2.94 | 1.83 | -5.39 | | -4.11 |
| C72S/Y12A | 68.77 | 21.53 | 2.47 | 1.37 | -0.47 | | -4.57 |
| C72S/F16A | 83.55 | 26.70 | 3.55 | 2.21 | 1.08 | | -3.73 |
| C72S/Y20A | 71.97 | 22.67 | 2.73 | 1.57 | -0.82 | | -4.37 |
| C72S/Y32A | 70.08 | 22.25 | 2.82 | 1.70 | 0.10 | | -4.24 |
| C72S/F46A | 177.22 | 57.76 | 8.65 | 5.81 | 5.82 | | -0.13 |
| C72S/W53A | 172.37 | 56.94 | 9.17 | 6.41 | 0.52 | | 0.47 |
| C72S/D6A | 157.57 | 50.81 | 7.14 | 4.62 | -2.03 | | -1.32 |
| C72S/E8A | 168.67 | 53.53 | 6.78 | 4.08 | -0.36 | | -1.86 |
| C72S/E24A | 135.46 | 43.74 | 6.19 | 4.03 | -0.59 | | -1.91 |
| C72S/R28A | 107.22 | 34.05 | 4.33 | 2.62 | -1.86 | | -3.32 |
| C72S/R29A | 145.96 | 46.28 | 5.82 | 3.49 | 1.49 | | -2.45 |
| C72S/K36A | 145.80 | 46.46 | 6.06 | 3.72 | 0.23 | | -2.22 |
| C72S/E67A | 149.80 | 48.81 | 7.30 | 4.90 | 1.24 | | -1.04 |
| C72S/D6A/R29A | 125.28 | 40.60 | 5.87 | 3.87 | -1.42 | | -2.07 |
| C72S/R28A/E67A | 110.04 | 36.61 | 6.11 | 4.35 | 0.24 | | -1.59 |

^a^ The free energy difference (ΔΔG_u_) was calculated to understand the effects of mutation on protein stability.. The ΔΔG_u (mutant － C72S)_ value is defined as ΔG_u (mutant)_ – ΔG_u (C72S)_, indicating the unfolding free energy change due to mutation. If the sign is negative, it indicates that the mutation destabilizes the protein, whereas the positive sign corresponds to the stabilization by the mutation. Mutations at the hydrophobic core 1 have the negative ΔΔG_u_, thus destabilization of the entire protein.
