# Supplementary material for: Residue-Specific Annotation of Disorder-to-Order Transition and Cathepsin Inhibition of a Propeptide-Like Crammer from D. melanogaster
Source: PLoS One. 2013 Jan 21;8(1):e54187. doi: 10.1371/journal.pone.0054187 (PMC3551606; doi:10.1371/journal.pone.0054187)
Supplement: Table S2 — Analysis of the π-π interactions of crammer and the propeptides of human cathepsins L (HCTSL), K (HCTSK) and S (HCTSS). (DOCX) [file pone.0054187.s010.docx]

**Table S2. Analysis of the π-π interactions of crammer and the propeptides of human cathepsins L (HCTSL), K (HCTSK) and S (HCTSS).**

| **Protein**  **(PDB code)^a^** | **Interaction** | **Distance (Å)**  **(centroid-centroid)** | **Dihedral Angle**  **(°)** |
| --- | --- | --- | --- |
| Crammer | Trp9-Try12 | 5.86 | 44.22 |
| (2KTW) | Trp9-Phe16 | 4.96 | 163.11 |
|  | Trp9-Try20 | 5.63 | 151.97 |
|  | Trp9-Try32 | 5.14 | 97.60 |
|  | Tyr12-Phe16 | 5.31 | 122.33 |
| HCTSL | Trp12-Trp15 | 5.68 | 171.14 |
| (1CS8) | Trp12-Tyr23 | 6.11 | 5.77 |
|  | Trp12-Trp35 | 5.34 | 55.03 |
|  | Trp15-Trp35 | 6.43 | 127.39 |
| HCTSK | Trp11-Try14 | 6.49 | 19.95 |
| (1BY8) | Trp11-Trp22 | 5.57 | 23.14 |
|  | Trp11-Try35 | 6.82 | 109.29 |
|  | Trp14-Try35 | 6.21 | 128.37 |
| HCTSS | Trp13-Trp16 | 5.84 | 165.22 |
| (2C0Y) | Trp13-Tyr24 | 5.88 | 172.33 |
|  | Trp13-Try37 | 5.34 | 59.30 |
|  | Trp16-Try20 | 5.49 | 128.41 |
|  | Tyr16-Trp37 | 6.24 | 128.78 |

^a^The 3D coordinates used for the calculations were taken from PDB entries 2KTW [[1](#_ENREF_1)], 1CS8 [[2](#_ENREF_2),[3](#_ENREF_3)], 1BY8 [[4](#_ENREF_4)], and 2C0Y [[5](#_ENREF_5)].
