# Supplementary material for: Residue-Specific Annotation of Disorder-to-Order Transition and Cathepsin Inhibition of a Propeptide-Like Crammer from D. melanogaster
Source: PLoS One. 2013 Jan 21;8(1):e54187. doi: 10.1371/journal.pone.0054187 (PMC3551606; doi:10.1371/journal.pone.0054187)
Supplement: Table S3 — Hydrophobic contact analysis. The software, Ligplot, [66] was used to analyze the hydrophobic contacts for five conserved aromatic residues (W9, Y12, F16, Y20, and Y32). These residues play an important role in the stabilization of the hydrophobic core 1 of crammer. (DOCX) [file pone.0054187.s011.docx]

**Table S3.** **Hydrophobic contact analysis. The software, Ligplot, [**[**7**](#_ENREF_7)**] was used to analyze the hydrophobic contacts for five conserved aromatic residues (W9, Y12, F16, Y20, and Y32). These residues play an important role in the stabilization of the hydrophobic core 1 of crammer.**

|  | **Aromatic residues in the hydrophobic core 1 of crammer^a^** | | | | |
| --- | --- | --- | --- | --- | --- |
|  | **W9** | **Y12** | **F16** | **Y20** | **Y32** |
| Hydrophobic Contacts | E8 | W9 | Y12 | W9 | V4 |
|  | V10 | E11 | K13 | K18 | E8 |
|  | Y12 | K13 | K15 | N19 | W9 |
|  | K13 | F16 | F16 | E21 | I31 |
|  | Y20 | I57 | D17 | D25 | A33 |
|  | Y32 |  | H59 | R28 | S35 |
|  | I57 |  |  | L63 | A61 |
|  | H59 |  |  |  |  |
|  | D62 |  |  |  |  |

^a^ The 3D coordinates used for the calculation were taken from PDB entry 2KTW [[2](#_ENREF_2)].
