# Supplementary material for: Residue-Specific Annotation of Disorder-to-Order Transition and Cathepsin Inhibition of a Propeptide-Like Crammer from D. melanogaster
Source: PLoS One. 2013 Jan 21;8(1):e54187. doi: 10.1371/journal.pone.0054187 (PMC3551606; doi:10.1371/journal.pone.0054187)
Supplement: Table S4 — The predicted helical propensities of double mutants in salt bridges by the agadir program ( http://agadir.crg.es ). (DOCX) [file pone.0054187.s012.docx]

**Table S4. The predicted helical propensities of double mutants in salt bridges by the agadir program (****http://agadir.crg.es).**

| **Mutants of salt bridges** | **C72S/D6A** | **C72S/E8A** | **C72S/E24A** | **C72S/R28A** | **C72S/R29A** | **C72S/K36A** | **C72S/E67A** |
| --- | --- | --- | --- | --- | --- | --- | --- |
| **Helical Content [%] pH 4.0** | **3.98** | **3.98** | **3.24** | **2.24** | **3.4** | **3.65** | **3.83** |
| **Helical Content [%] pH 6.0** | **11.65** | **11.67** | **8.92** | **5.41** | **8.8** | **6.95** | **8.49** |
